# Transcriptomic and physiological analyses revealed nicotianamine enhances wheat tolerance to excess manganese

**DOI:** 10.1016/j.isci.2025.113671

**Published:** 2025-10-03

**Authors:** Daozhen Luo, Qing Li, Fei Pang, Wenjie Zhang, Muhammad Usman, Yangrui Li, Yongxiu Xing, Dengfeng Dong

**Affiliations:** 1 Guangxi Key Laboratory of Agro-Environment and Agric-Products Safety, College of Agriculture, Guangxi University, Nanning 530004, China; 2 Sugarcane Research Institute, Guangxi Academy of Agricultural Sciences, Nanning 530007, China

**Keywords:** Plant Biology, Agricultural science, Agricultural soil science, Agricultural plant products

## Abstract

Manganese (Mn) is an essential plant micronutrient but toxic at supra-optimal concentrations. Although nicotianamine (NA) biosynthesis is metal inducible, its specific role in Mn detoxification in wheat remains poorly characterized. Integrated transcriptomic and physiological analyses of Mn-tolerant (ET8, Carazinho) and Mn-sensitive (ES8, Egret) wheat near-isogenic lines and their parental cultivars under excess Mn revealed pronounced upregulation of genes encoding nicotianamine synthase (*TaNAS*), nicotianamine aminotransferase (*TaNAAT*), and putative Mn-NA transporters (*TaYSL2*, *TaYSL6*) in tolerant genotypes. This transcriptional response correlated with elevated NA accumulation in roots under Mn stress. Exogenous NA application enhanced Mn tolerance in hydroponically grown seedlings in a concentration-dependent manner, accompanied by reduced tissue Mn accumulation, and maintained homeostasis of essential cations (Ca, Mg, Fe, Zn, Cu) in both roots and shoots. Our findings demonstrate that NA biosynthesis and transport contribute to Mn tolerance by modulating Mn partitioning and metal ion homeostasis. We further propose candidate genes (*TaNAS*, *TaNAAT*, *TaYSL2*, *TaYSL6*) as potential targets for breeding Mn-tolerant wheat.

## Highlights

• Mn tolerance relies on nicotianamine (NA) biosynthesis gene induction in wheat
• Mn-tolerant wheat genotypes elevate NA production for superior Mn sequestration
• Exogenous NA alleviates toxicity by reducing Mn uptake and restoring homeostasis

## Graphical abstract

**Figure:**
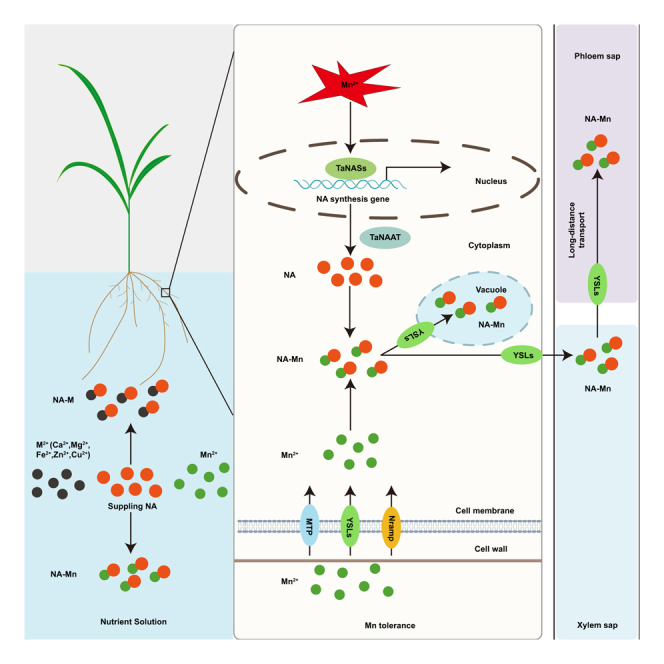

## Introduction

Mn is an essential micronutrient for plant growth and development and plays a crucial role in various processes throughout a plant’s life cycle. It acts as a cofactor for enzymes and a catalyst in biological metal clusters, contributing to essential functions such as photosynthesis, respiration, and scavenging reactive oxygen species (ROS), pathogen defense, and hormone signaling.^1^ Mn toxicity typically manifests in poorly drained, strongly acidic soils (pH < 5.5), where reducing conditions and high proton activity significantly enhance Mn^2+^ bioavailability.^2^ Beyond pH and redox status, the bioavailability of other soil solutes influences Mn dynamics through ion antagonism and/or by altering its speciation within the rhizosphere. For instance, alkaline earth metal cations such as Mg^2+^ and Ca^2+^ not only regulate soil acidity but also compete with Mn^2+^ for plant uptake.^3^ Crucially, Mn toxicity thresholds exhibit significant species- and genotype-specific variability.^1^^,^^4^ While most plants maintain optimal tissue Mn concentrations between 20 and 40 mg/kg dry weight, tolerant species (e.g., *Oryza sativa* L. and *Eucalyptus globulus* Labill.) can withstand tissue Mn concentrations exceeding 500 mg/kg—levels that are lethal to sensitive crops like wheat (*Triticum aestivum* L.).^5^ Symptoms of Mn toxicity include chlorosis in young leaves, dark necrotic spots on mature leaves, and crinkled leaves, ultimately inhibiting plant growth.^6^ Mn toxicity can disrupt various physiological processes in plant cells, such as triggering oxidative stress, causing lipid peroxidation, inhibiting enzyme activity, impairing chlorophyll biosynthesis and photosynthesis, and disturbing the uptake and translocation of other mineral elements.^3^

Plants have developed several strategies to cope with Mn toxicity, including enhancing the activity of antioxidant enzymes, regulating organic acid (OA) metabolism, controlling ion absorption, and differentially compartmentalizing Mn at the subcellular level.^5^ For example, Mn toxicity increases the activities of ascorbate peroxidase and guaiacol peroxidase, thus enhancing Mn tolerance in ryegrass.^7^ The superior Mn tolerance of *Stylosanthes guianensis* is achieved by coordinating internal and external Mn detoxification through malate synthesis and exudation.^8^ Sequestration and compartmentalization of Mn in subcellular sites are crucial for conferring Mn tolerance.^9^ Additionally, roots can enhance tolerance to Mn toxicity by regulating the uptake of mineral nutrients through the control of metal transporter genes.^10^

Plants maintain Mn homeostasis through the activities of several transporters from diverse metal transporter families.^6^ In rice, OsNRAMP5 (natural resistance-associated macrophage protein 5) mediates the uptake and translocation of Mn^2+^ in conjunction with OsMTP9 (metal tolerance protein 9).^11^^,^^12^ Two *Arabidopsis* ZIP (zinc-regulated transporter/iron-regulated transporter-like protein) family members, AtZIP1 and AtZIP2, are involved in Mn translocation from roots to shoots.^13^ Yellow stripe-like transporters (YSLs) are involved in the uptake, transport, and relocation of metal complexes, such as Mn- nicotianamine (NA) to maintain Mn ion balance in plants.^14^^,^^15^ The vacuolar-localized Mn transporters AtMTP8 and AtVIT1 (vacuolar iron transporter 1) are responsible for Mn^2+^ sequestration into vacuoles.^16^^,^^17^ Most Mn transporters are not only specific to the metal but also affect the homeostasis of other minerals, such as calcium (Ca), magnesium (Mg), iron (Fe), zinc (Zn), and copper (Cu).^1^ Multiple transporter families, including YSL,^15^^,^^18^ MTP,^17^^,^^19^ Nramp,^12^^,^^20^^,^^21^ and VIT,^16^^,^^22^ compete for Mn^2+^ binding alongside other divalent cations (Ca^2+^, Mg^2+^, Fe^2+^, Zn^2+^, Cu^2+^), thereby creating complex cross-homeostasis effects under Mn stress.

NA, a low-molecular-weight nitrogen-containing metal-binding ligand, exhibits organ-specific accumulation patterns in plants under metal deficiency or excess. It is crucial for maintaining plant metal ion homeostasis and scavenging ROS.^23^ Synthesized from S-adenosylmethionine by nicotianamine synthase (NAS), NA is further converted to mugineic acids (MAs) via nicotianamine aminotransferase (NAAT).^24^ Its biosynthesis is *in vivo* induced by multiple metals. As a non-proteinogenic amino acid, NA primarily functions in detoxifying and transporting Fe, Mn, Zn, Cd, and Cu. Previous studies have highlighted NA’s role in Fe and Zn biofortification^25^ and the detoxification of Mn,^26^ Cd,^27^ and Cd/Zn,^28^ underscoring its importance in regulating metal homeostasis and detoxification.^23^

As the world’s most widely cultivated staple crop,^29^ wheat’s response to Mn stress warrants particular attention. Although NA’s alleviatory effects on Mn toxicity are established, its specific functions in wheat under Mn stress remain undefined. Our study revealed distinct Mn accumulation and distribution patterns among wheat genotypes varying in Mn tolerance. Transcriptomic analysis identified significant upregulation of NA biosynthetic pathways under Mn stress, which was confirmed by physiological and biochemical assays showing stress-induced enhancement of endogenous NA production. Critically, we demonstrated that exogenous NA application alleviates Mn toxicity by modulating Mn and other essential metal ion homeostasis. This work elucidates the functional role of NA in mediating Mn detoxification and metal ion homeostasis, providing mechanistic insights into wheat Mn tolerance.

## Results

### Gene expression profile in wheat roots under Mn stress

**Figure 1:**
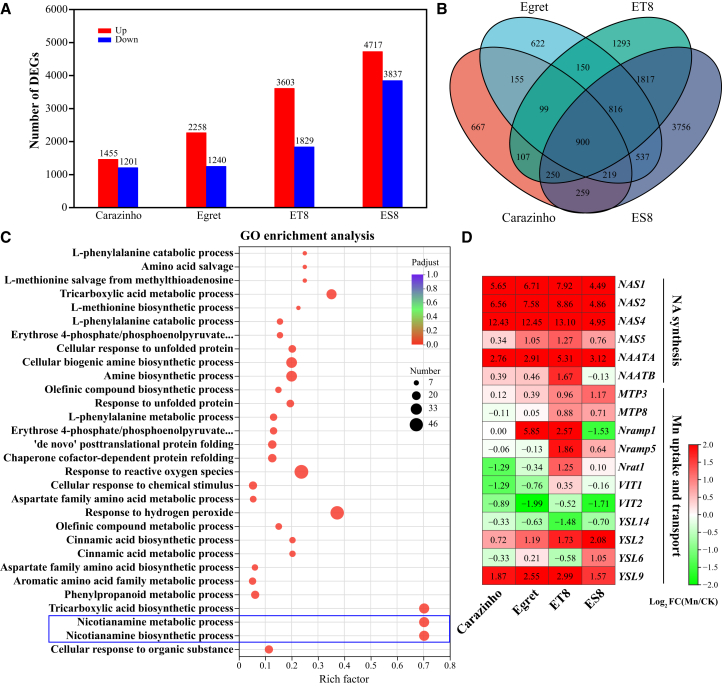
Gene expression profile in wheat roots under Mn stress (A) Number of DEGs in four wheat genotypes. (B) Venn diagram of DEGs in four genotypes of wheat. (C) GO enrichment analysis of common DEGs. (D) Heatmap of DEGs related to NA biosynthesis and Mn uptake and transport.

RNA sequencing (RNA-seq) analysis was performed on roots of four wheat genotypes (Carazinho, Egret, ET8, and ES8) under Mn stress. Comparing Mn-treated roots to controls (CK) identified 2,656 differentially expressed genes (DEGs) in Carazinho (1,455 upregulated, 1,201 downregulated), 2,498 DEGs in Egret (2,258 upregulated, 1,240 downregulated), 5,432 DEGs in ET8 (3,603 upregulated, 1,829 downregulated), and 8,554 DEGs in ES8 (4,717 upregulated, 3,837 downregulated) (fold change >2, false discovery rate [FDR] < 0.01; Figure 1A; Table S1). Among these, 900 DEGs were common to all four genotypes, indicating a shared transcriptional response to Mn stress (Figures 1B; Table S1). Gene ontology (GO) enrichment analysis of these common DEGs further revealed significant enrichment for the NA metabolic process (GO:0030417) and NA biosynthetic process (GO:0030418) (Figure 1C). Collectively, these results demonstrate that Mn stress induces numerous DEGs involved in Mn uptake and transport and highlight DEGs related to NA biosynthesis and transport as key contributors to Mn homeostasis (Figure 1D).

**Figure 2:**
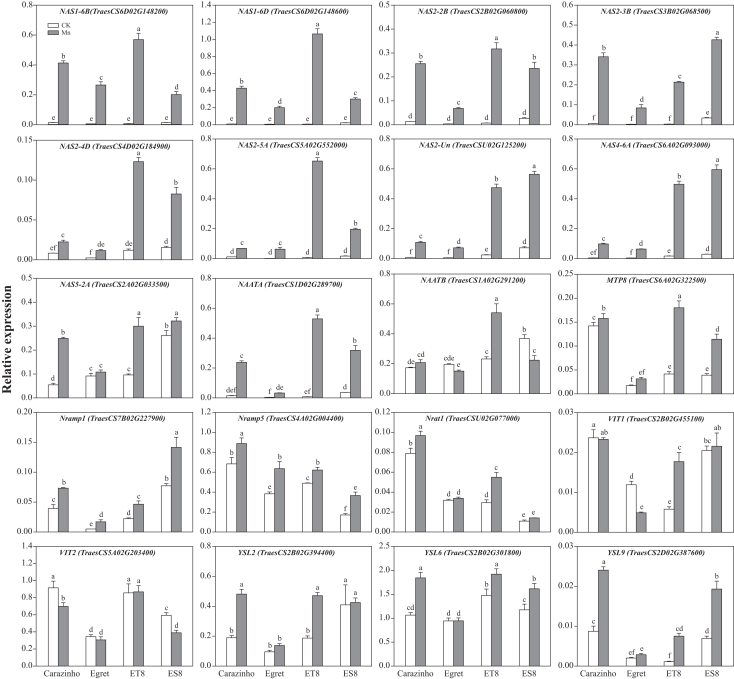
RT-qPCR validation of induction of the NA biosynthesis and Mn transporter genes *TaActin* served as the internal control. Data are represented as mean ± SD (*n* = 3 biological replicates). Bars with different lowercase letters indicate significant differences among treatments (*p* < 0.05, one-way ANOVA with Duncan’s multiple range test).

Therefore, RT-qPCR was performed to validate the RNA-seq results. The RT-qPCR data confirmed the transcriptome findings, demonstrating that the expression levels of *TaNAS* genes (*TaNAS1*, *TaNAS2*, *TaNAS4*, and *TaNAS5*) and *TaNAAT* genes (*TaNAATA* and *TaNAATB*) were significantly upregulated under Mn stress (Figure 2), notably in ET8. Additionally, RT-PCR measured the expression of Mn uptake/transport genes: the tonoplast-localized Mn transporter *TaMTP8* was significantly upregulated in Carazinho, ET8, and ES8 (but not in Egret), with higher levels in tolerant (Carazinho and ET8) vs. sensitive (Egret and ES8) genotypes; the high-affinity Mn transporters *TaNramp1* and *TaNramp5* were also upregulated; the root plasma membrane Al uptake transporter TaNrat1 was upregulated in Carazinho and ET8, while remaining unchanged in Egret and ES8; the vacuolar Fe/Mn transporters *TaVIT1* and *TaVIT2* exhibited divergent patterns—*TaVIT1* was significantly upregulated in ET8, downregulated in Egret, and unchanged in Carazinho and ES8, while *TaVIT2* displayed the opposite trend; finally, the metal-NA transporters *TaYSL2*, *TaYSL6*, and *TaYSL9* were also upregulated, particularly in the tolerant genotypes Carazinho and ET8.

### Tolerant genotypes limit Mn translocation to shoots

**Figure 3:**
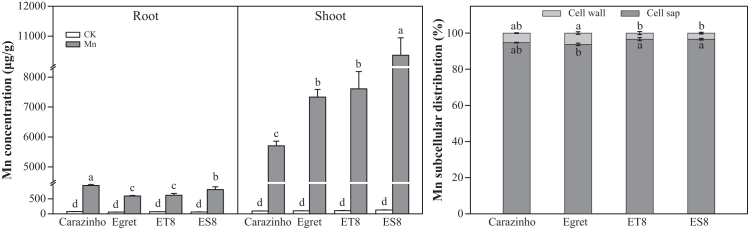
Mn concentration and subcellular distribution in root cells Data are represented as mean ± SD (*n* = 3 biological replicates). Bars with different lowercase letters indicate significant differences among treatments (*p* < 0.05, one-way ANOVA with Duncan’s multiple range test).

To investigate Mn absorption and translocation in wheat, we analyzed the subcellular distribution of Mn in root tips and its accumulation in roots and shoots under Mn stress. Results showed that >93% of Mn was localized to the cell sap of root tip cells, with only a minor fraction associated with cell walls (Figure 3). When exposed to 500 μM Mn in the nutrient solution, Mn concentrations significantly increased in both shoots and roots. Notably, shoot Mn concentrations exceeded those in roots.

Comparisons between near-isogenic lines revealed significantly lower shoot Mn concentrations in the tolerant genotype ET8 (7,607.8 μg/g) compared to its sensitive counterpart ES8 (10,361.5 μg/g). Similarly, the non-recurrent parent (tolerant cultivar Carazinho) exhibited significantly lower shoot Mn (5,704.9 μg/g) than the recurrent parent (sensitive cultivar Egret; 7,332.5 μg/g) (Figure 3).

### Mn stress induced endogenous NA synthesis but not exudation

**Figure 4:**
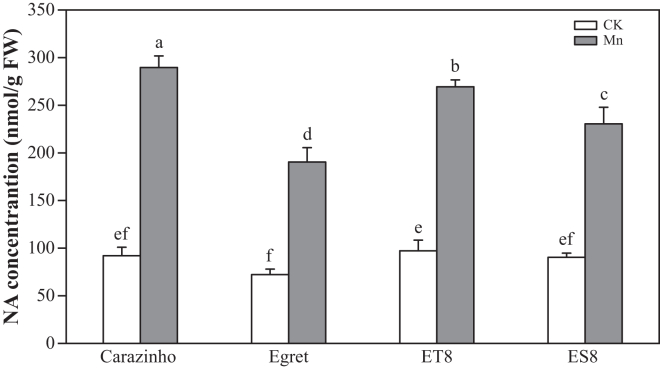
Endogenous NA accumulation in roots under Mn stress Data are represented as mean ± SD (*n* = 3 biological replicates). Bars with different lowercase letters indicate significant differences among treatments (*p* < 0.05, one-way ANOVA with Duncan’s multiple range test).

Consistent with the transcriptomic and RT-qPCR analysis showing enhanced expression of multiple *NAS* genes was in Mn-stressed wheat roots as compared to the control (Figure 2), endogenous NA concentrations in wheat roots significantly increased under Mn stress. Concentrations increased 3.1-fold in Carazinho, 2.6-fold in Egret, 2.8-fold in ET8, and 2.5-fold in ES8 compared to controls (Figure 4). Under Mn stress, the tolerant varieties (Carazinho and ET8) accumulated higher NA concentrations than the sensitive varieties (Egret and ES8). ET8 exhibited a significantly higher NA concentration than Egret, with no significant differences observed among the other varieties.

Despite stimulating endogenous NA synthesis and accumulation, Mn stress did not induce detectable NA exudation from roots.

### Exogenous NA conferred wheat tolerance to Mn stress

**Figure 5:**
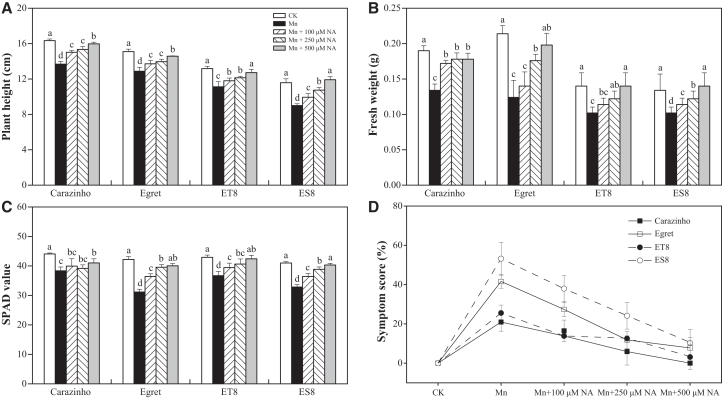
Phenotypic responses of wheat seedlings to Mn stress and NA supplementation (A) Plant height. (B) Fresh weight. (C) SPAD chlorophyll index. (D) Mn toxicity symptom score. Data are represented as mean ± SD (*n* = 3 biological replicates). Bars with different lowercase letters indicate significant differences among treatments (*p* < 0.05, one-way ANOVA with Duncan’s multiple range test).

Under excess Mn conditions, wheat seedlings exhibited significant growth inhibition. Compared to the control (CK), cultivars Carazinho, Egret, ET8, and ES8 exhibited reductions in plant height, fresh weight, and soil and plant analyzer development (SPAD) values as follows: 16.3%, 26.2%, and 13.0% for Carazinho; 14.7%, 42.1%, and 26.2% for Egret; 15.6%, 27.1%, and 14.5% for ET8; and 22.3%, 23.9%, and 19.9% for ES8 (Figures 5A–5C). Mn toxicity symptoms, including leaf chlorosis, necrotic lesions, and leaf crinkling, were visually observed (Figure S1). The severity of Mn poisoning was quantified using a symptom score, defined as the percentage of leaves showing Mn toxicity symptoms. The percentages of affected leaves were 20.9% (Carazinho), 41.6% (Egret), 25.5% (ET8), and 53.2% (ES8), confirming the phenotypic sensitivity gradient: ES8 > Egret > ET8 > Carazinho (Figure 5D).

To evaluate the effect of exogenous NA on Mn tolerance, seedlings were treated with 100, 250, or 500 μM NA for 6 days under excess Mn conditions. Exogenous NA significantly alleviated Mn toxicity in a dose-dependent manner across all cultivars, with the most pronounced effects observed at 500 μM (Figure S1). Plant height recovered to 14%–33% above Mn-stressed levels, fresh weight increased 1.4- to 1.9-fold, and chlorophyll content recovered to 89%–99% of control levels (Figures 5A–5C). At 500 μM NA, symptom scores of the four cultivars dropped sharply to ≤10.3% from initial values of 20.9%–53.2% (Figure 5D).

### Exogenous NA suppressed Mn and restored ion homeostasis

**Figure 6:**
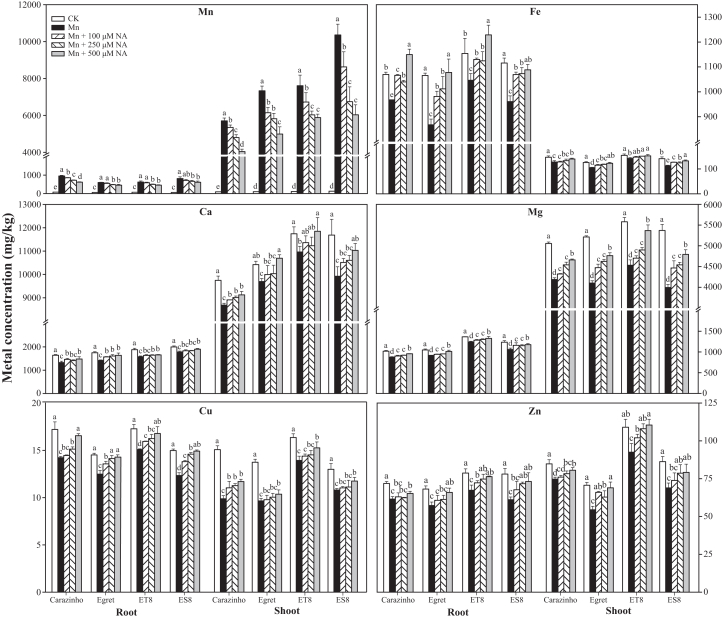
Concentrations of Mn, Fe, Ca, Mg, Cu, and Zn in wheat under Mn stress and NA supplementation Data are represented as mean ± SD (*n* = 3 biological replicates). Bars with different lowercase letters indicate significant differences among treatments (*p* < 0.05, one-way ANOVA with Duncan’s multiple range test).

Mn concentrations in roots and shoots were quantified to confirm whether exogenous NA reduces Mn uptake and accumulation in wheat (Figure 6). Exogenous NA significantly decreased Mn concentrations in both roots and shoots across all four wheat genotypes, with maximal reduction observed at 500 μM NA. Mn concentrations in the roots decreased by 34.7% (Carazinho), 24.6% (Egret), 26.8% (ET8), and 24.5% (ES8), and in the shoots, by 29.2% (Carazinho), 32.0% (Egret), 22.7% (ET8), and 41.8% (ES8).

Under Mn stress, wheat cultivars exhibited distinct patterns of metal accumulation: Fe displayed root-preferential distribution (867.5–1,228.7 μg/g DW (dry weight) in roots vs. 105.0–152.5 μg/g DW in shoots), while Ca (1,642.4–1,997.6 μg/g DW in roots vs. 8,687.2–11,742.3 μg/g DW in shoots) and Mg (875.6–1,365.3 μg/g DW in roots vs. 3,996.6–5,580.1 μg/g DW in shoots) predominantly accumulated in shoots. Micronutrients Cu (12.3–17.3 μg/g DW in roots vs. 9.7–16.4 μg/g DW in shoots) and Zn (61.1–78.7 μg/g DW in roots vs. 54.5–110.5 μg/g DW in shoots) showed cultivar-specific variations, with ET8 maintaining the highest Zn levels. NA application (100–500 μM) dose dependently mitigated these alterations, most effectively at 500 μM: reducing Mn accumulation by 24.2%–47.9%, restoring Fe to 1,077.6–1,228.7 μg/g DW in roots, increasing shoot Ca to 9,009.9–11,849.2 μg/g DW, improving Mg to 4,542.6–5,367.7 μg/g DW in shoots, and enhancing Cu/Zn status to 14.9–16.8/68.9–110.5 μg/g DW, respectively.

## Discussion

### Manganese accumulation and translocation in wheat

Excessive Mn can adversely affect plant growth and development, exhibiting significant species and genotype specificity.^3^^,^^4^ Rice (*Oryza sativa* L.)^12^^,^^14^^,^^19^^,^^30^ and *Stylosanthes guianensis*^8^ exhibit notably higher Mn tolerance than wheat (*Triticum aestivum* L.), attributed to its enhanced Mn transport capacity. Mn toxicity universally impairs plant growth by reducing biomass accumulation, chlorophyll content, and photosynthetic efficiency.^1^ Exposure to 500 μM Mn triggered severe toxicity symptoms, including biomass reduction, chlorophyll degradation, and inhibition of photosynthesis. Notably, shoot Mn concentrations exceed root levels by 10-fold, accompanied by visible symptoms such as leaf chlorosis, curling, and necrotic spots, particularly in young leaves. Further analysis revealed that 93.76% of absorbed Mn was localized in the root tip cell sap, with only a minimal fraction (<0.634%) bound to cell walls (Figure 3), suggesting limited apoplastic retention and efficient translocation to aerial parts. Contrastingly, in peanut (*Arachis hypogaea* L.), Mn toxicity induces characteristic stress symptoms, including Mn speckles and leaf wrinkling.^10^ Mechanistically, these symptoms are linked to oxidative stress resulting from diminished ROS scavenging capacity,^31^^,^^32^ with necrotic spot formation potentially arising from chloroplast damage and elevated free oxygen radicals due to suppressed MnSOD activity.^33^ However, plants employ distinct strategies for Mn detoxification. Unlike wheat, which showed preferential Mn accumulation in shoots, peanuts display a root-biased distribution pattern, with significantly higher Mn concentrations in roots than in leaves.^10^ This tissue-specific partitioning suggests that species have species-specific Mn sequestration strategies. Genotypic variation in Mn tolerance is well documented across various crops, including wheat,^34^ soybean,^35^ and barley,^4^^,^^32^ with significant intraspecific differences being observed. Our study of near-isogenic wheat lines revealed contrasting Mn accumulation patterns: ET8 accumulated significantly less Mn in shoots (7,607.8 μg/g) than ES8 (10,361.5 μg/g). Similarly, the non-recurrent parent cultivar Carazinho exhibited lower shoot Mn concentration (5,704.9 μg/g) compared to the recurrent parent Egret (7,332.5 μg/g). These findings, along with observed interspecific phenotypic variation, underscore the critical role of genetic factors in Mn tolerance.^3^ Nevertheless, the molecular mechanisms underlying these genotypic differences in Mn detoxification remain poorly understood.

### Nicotianamine biosynthesis enhances Mn tolerance in wheat

NA, a low-molecular-weight metal chelator, plays a pivotal role in maintaining the homeostasis of essential micronutrients, including Fe, Zn, and Mn.^23^ It protects plant cells from metal-induced oxidative damage.^36^ Our transcriptomic analysis identified significant enrichment of NA biosynthetic and metabolic processes (GO:0030418, GO:0030417) among the common DEGs in wheat subjected to Mn stress (Figure 1C). Notably, key genes involved in NA biosynthesis (*TaNAS1*, *TaNAS2*, *TaNAS4*, *TaNAS5*, and *TaNAAT*) were significantly upregulated in roots (Figure 1D), suggesting a critical role for these genes in Mn tolerance in wheat. Similar findings have been reported in *Sedum alfredii*; *SaNAS1* overexpression enhanced tolerance to Cd and Zn.^28^ In *Arabidopsis*, NA accumulation correlated with *TaNAS-D* expression.^37^ The *TaNAS-D* transgenic *Arabidopsis* plants exhibited lower malondialdehyde levels and higher activities of peroxidase, superoxide dismutase, and catalase, thereby reducing membrane injury.^37^ Consistent with these observations, NA levels in wheat roots increased more than 3-fold under Mn stress, with tolerant genotypes (Carazinho, ET8) exhibiting higher *TaNAS* expression and NA accumulation than sensitive ones (Egret, ES8) (Figure 4). This differential response provides evidence that enhanced NA biosynthesis serves as a key physiological mechanism in Mn stress tolerance. Specifically, the elevated NA production in tolerant genotypes facilitates more efficient Mn chelation and detoxification, thereby mitigating the deleterious effects of Mn toxicity.^37^ The critical role of NA in metal detoxification is further supported by studies in *Arabidopsis halleri*, a plant species that accumulates zinc. Exogenous NA application similarly decreased zinc uptake in *Arabidopsis* and alleviated toxicity symptoms, mirroring the protective role of NA in metal stress adaptation. Consistent with these observations, the NA application dramatically alleviated Mn toxicity, restoring growth parameters to near-control levels (89%–99% recovery) and reducing visible symptoms from 20.9%–53.2% to ≤10.3% when the application reaches 500 μM (Figure 5). These findings collectively highlight NA’s dual role in chelating excess Mn and maintaining homeostasis in wheat.

Although exogenous NA alleviates Mn toxicity in wheat, NA secretion was undetectable in root exudates. In rice, two genes (*ENA1* and *ENA2*) putatively encoding NA efflux transporters have been identified, yet their biological functions and involvement in NA secretion remain uncharacterized.^38^^,^^39^ Graminaceous plants have evolved a unique strategy for iron acquisition through the secretion of MA family phytosiderophores (PSs).^40^ Notably, compounds including MA, deoxymugineic acid (DMA), hydroxymugineic acid, avenic acid, and hydroxyavenic acid were detected in Fe-deficient barley root exudates, whereas neither NA nor its biosynthetic intermediate 3″-oxo acid were detected.^41^ These findings imply that NA might be metabolized prior to secretion in graminaceous roots. We hypothesize that under Mn stress, NA in wheat roots is rapidly converted into MAs prior to secretion, as supported by the significant upregulation of *TaNAAT*— a key gene encoding NAAT that catalyzes the conversion of NA to DMA precursors.^38^ If secreted as Mn-DMA complexes, these chelates could potentially be imported via YSL transporters, which display binding affinity for metal-MA chelates including Mn-DMA.^42^

### NA improves element uptake and accumulation in wheat

Nutrient elements such as Ca, Mg, Fe, Mn, Cu, and Zn play pivotal roles in multiple physiological processes in plants, including growth promotion, enzyme activation, photosynthesis facilitation, and redox balance maintenance,^43^^,^^44^ and engage in competitive binding with transporters and chelators. This competitive interaction serves as a crucial mechanism to reduce Mn uptake and mitigate the oxidative damage caused by excessive Mn accumulation.^1^^,^^2^ Mn detoxification is a highly regulated process that requires precise control over Mn uptake, transport, and distribution within the plant to prevent detrimental effects.^45^

The YSL family transporters act as competitive metal-binding proteins. They are essential for maintaining Mn homeostasis by mediating the transport of Mn-NA complexes. Meanwhile, their activity also exerts a significant influence on the uptake and distribution of other essential metals.^15^^,^^18^ For example, HvYSL2 transports PS complexes with various metal ions, including Fe^3+^, Zn^2+^, Ni^2+^, Cu^2+^, Mn^2+^, and Co^2+^. Besides the YSL family, other transporter families, namely MTP8,^17^^,^^19^ Nramp,^12^^,^^20^^,^^21^ and VIT,^16^^,^^22^ display competitive binding affinities for Mn^2+^ and other divalent cations (Ca^2+^, Mg^2+^, Fe^2+^, Zn^2+^, Cu^2+^), creating complex cross-homeostasis effects under Mn stress. In tolerant wheat genotypes, the differential upregulation of these transporter genes such as *TaYSL2/6*, *TaMTP8*, *TaNramp1/5*, and *TaVIT1/2* indicates their crucial role in competitive allocation of metals. This competition also extends to the chelation system. An increase in NA synthesis, driven by the upregulation of *TaNAS*/*TaNAAT* genes, leads to the production of more metal-chelating ligands. These ligands preferentially form Mn-NA complexes, which in turn reduces the competition from free Mn^2+^ for other metal transporters, thereby facilitating the uptake of essential nutrients (Figure 6).

In summary, the coordinated interplay among transporters with overlapping metal specificities, chelators with differential metal affinities, and compartmentalization systems constitutes a sophisticated regulatory network for cellular metal homeostasis under Mn toxicity.^1^^,^^45^ This regulatory mechanism provides a plausible explanation for the observed metal redistribution after NA treatment. Specifically, NA treatment results in increased concentrations of Fe, Zn, and Cu, accompanied by decreased Mn accumulation, which is consistent with the changes in metal ratios reported in *OsNAS*/*OsNAAT*-expressing rice.^46^^,^^47^ In *Arabidopsis*, studies have shown that excessive Zn leads to reduced shoot Fe content. However, exogenous application of NA can modulate the expression of *IRT1* and *FRO2*, decreasing Zn accumulation while increasing shoot Fe levels.^48^ The promiscuous substrate specificity of these metal-binding proteins establishes an intricate competitive network that governs the final metal allocation in plants under Mn stress.

### Conclusions

This study demonstrates that NA biosynthesis and transport play a crucial role in Mn detoxification in wheat. Integrated transcriptomic and physiological analyses revealed pronounced upregulation of NA biosynthesis and Mn-NA transporter genes (*TaNAS*, *TaNAAT*, *TaYSL2*, *TaYSL6*) in Mn-tolerant genotypes under excess Mn, leading to enhanced NA accumulation and reduced Mn toxicity. Exogenous NA application alleviated Mn stress by modulating Mn partitioning and maintaining essential metal ion homeostasis. We propose that NA mediates Mn detoxification by (1) facilitating Mn-NA chelation for vacuolar sequestration and (2) mitigating Mn^2+^ competition with divalent cations. These findings nominate *TaNAS*, *TaNAAT*, and *TaYSLs* as candidate targets for breeding tolerant wheat cultivars.

### Limitations of the study

While this work reveals the role of NA in Mn tolerance, three key limitations remain. First, while qPCR confirmed stress-induced expression of NA pathway genes (*TaNAS/TaNAAT/TaYSLs*), functional validation via transgenic approaches (e.g., overexpression/knockout) was hindered by the recalcitrance of non-model wheat genotypes to established transformation protocols. Second, the proposed mechanism of NA-mediated Mn chelation and transport lacks direct evidence (e.g., Mn-NA complex quantification or subcellular localization). Third, experiments focused on hydroponically grown seedlings, limiting extrapolation to field conditions or mature plants. Future studies would (1) validate gene functions in transformable model wheats (e.g., cv. Fielder or Y158), (2) quantify Mn speciation (e.g., X-ray absorption spectroscopy) to confirm NA-Mn complex formation, and (3) assess NA efficacy in soil-based Mn toxicity scenarios.

## Resource availability

### Lead contact

Further information and requests for resources and reagents should be directed to and will be fulfilled by the lead contact, Dengfeng Dong (dongdfxy@gxu.edu.cn).

### Materials availability

This study did not generate new unique reagents.

### Data and code availability

• Data: The RNA-seq raw data is deposited in the NCBI Sequence Read Archive under the accession number NCBI: PRJNA1031207.
• Code: This paper does not report original code.
• Any additional information required to reanalyze the data reported in this paper is available from the lead contact upon request

## Acknowledgments

We gratefully acknowledge Shanghai Majorbio Bio-pharm Biotechnology Co., Ltd. (Shanghai, China) for providing high-throughput sequencing services and access to their comprehensive bioinformatics platform. This study was supported by the 10.13039/501100001809National Natural Science Foundation of China, China (grant no. 32460463) and the Guangxi Natural Science Foundation, China (grant no. 2023GXNSFAA026445).

## Author contributions

Conceptualization, D.D., Y.L., and Y.X.; methodology, D.L. and Q.L.; investigation, D.L., Q.L., F.P., and W.Z.; formal analysis, D.L. and Q.L.; writing – original draft, D.L. and Q.L.; writing – review & editing, M.U. and D.D.; supervision, Y.L.; project administration, D.D.; funding acquisition, D.D. All authors discussed results and approved the final manuscript.

## Declaration of interests

The authors declare no competing interests.

## Declaration of generative AI and AI-assisted technologies in the writing process

During the preparation of this work, the authors used Grammarly for language polishing and proofreading. The AI-assisted features were utilized solely to check and suggest improvements for grammar, spelling, and sentence clarity. All scientific content, data interpretation, and conclusions remain entirely generated by the authors. After using this tool, the authors thoroughly reviewed and edited the content as needed and take full responsibility for the content of the publication.

## STAR★Methods

### Key resources table

**Table:** 

REAGENT or RESOURCE	SOURCE	IDENTIFIER
**Biological samples**		

Seeds of Wheat near-isogenic lines (ET8, ES8) and parental cultivars (Carazinho, Egret)	CSIRO Plant Industry, Canberra, Australia	–

**Chemicals, peptides, and recombinant proteins**		

Calcium hypochlorite	Sinopharm Chemical	Cat#10020108
Potassium chloride	Aladdin	Cat# P112119
Magnesium chloride hexahydrate	Aladdin	Cat#M116097
Boric acid	Sangon Biotech	Cat#A500897
Zinc sulfate heptahydrate	Sinopharm Chemical	Cat#10024018
Copper sulfate pentahydrate	Aladdin	Cat# C111588
Sodium molybdate dihydrate	Sigma-Aldrich	Cat#331058
Ethylenediaminetetraacetic acid ferric sodium salt	Sigma-Aldrich	Cat#E6760
Nicotianamine	Toronto Research Chemicals	Cat# N408500

**Critical commercial assays**		

TRIzol™ Plus RNA Purification Kit	Invitrogen	Cat#12183555
PrimeScript™ RT Master Mix	Takara	Cat#RR036A
TB Green® Premix Ex Taq™ II	Takara	Cat#RR086B
Ultrafree-MC Centrifugal Filter	Millipore	Cat#UFC40HV00

**Deposited data**		

Transcriptome sequencing data	This article	PRJNA1031207

**Oligonucleotides**		

qRT-PCR primer, see Table S1	This article	–

**Software and algorithms**		

Majorbio Cloud	Ren et al.^49^	https://cloud.majorbio.com/
RSEM	Li and Dewey^50^	https://github.com/deweylab/RSEM
DESeq2	Love et al.^51^	https://bioconductor.org/packages/release/bioc/html/DESeq2.html
TBtools	Chen et al.^52^	https://github.com/CJ-Chen/TBtools
Goatools	Klopfenstein et al.^53^	https://github.com/tanghaibao/Goatools
KOBAS	Xie et al.^54^	ftp://ftp.cbi.pku.edu.cn/pub/KOBAS_3.0_DOWNLOAD/docker
SPSS 20.0	IBM	https://www.ibm.com/analytics/spss-statistics-software
Primer3plus	–	https://www.primer3plus.com/

### Method details

#### Plant materials and treatment

Seeds of near-isogenic wheat lines (over 95% genetic similarity) contrasting in Al tolerance at the *ALMT1* (*aluminum activated malate transporter 1*) locus were used: ET8 (homozygous Al-tolerant), and ES8 (homozygous Al-sensitive). The parental lines, Carazinho (Al tolerant) and Egret (Al sensitive), were also included. The ET8 and ES8 lines were developed through a cross between Carazinho and Egret, followed by eight successive backcrosses to Egret or Egret-derived lines. Consistent with our previous findings,^55^ Mn tolerance co-segregates with Al tolerance in these near-isogenic wheat lines (ET8 and ES8) and their parents (Carazinho and Egret). All seeds were kindly supplied by Dr. Peter R. Ryan (CSIRO Plant Industry, Canberra, Australia).

Wheat seeds were surface-sterilized with 0.5% (v/v) NaClO solution for 20 min, rinsed thoroughly with sterile water, and germinated on moistened filter paper at 4°C for 4–5 days. Seedlings were then transplanted into continuously aerated modified Magnavaca’s solution^56^ containing the following components: 1 mM KCl, 1.5 mM NH_4_NO_3_, 1 mM CaCl_2_·2H_2_O, 45 μM KH_2_PO_4_, 200 μM MgSO_4_·7H_2_O, 500 μM Mg(NO_3_)_2_·6H_2_O, 155 μM MgCl_2_·6H_2_O, 11.8 μM MnCl_2_·4H_2_O, 33 μM H_3_BO_3_, 3.06 μM ZnSO_4_·7H_2_O, 0.8 μM CuSO_4_·5H_2_O, 1.07 μM Na_2_MoO_4_·2H_2_O, and 77 μM EDTA-FeNa. Seedlings underwent a 2-day acclimation to low pH in a walk-in growth chamber under controlled conditions: 23°C, a 14 h photoperiod (400 μmol m^−2^ s^−1^ illumination), and 60–80% relative humidity. Following acclimation, stress treatments were initiated by replacing the fresh Magnavaca medium (pH 4.2) amended with different combinations of Mn (as MnCl_2_) and NA (Toronto Research Chemicals): control (CK, no additions), 500 μM MnCl_2_ alone, 500 μM MnCl_2_ + 100 μM NA, 500 μM MnCl_2_ + 250 μM NA, and 500 μM MnCl_2_ + 500 μM NA. pH was monitored daily and maintained at 4.2. Treatments lasted for 6 days and followed a completely randomized block design with at least three biological replicates per group.

#### Morphological parameters determination

After six days of treatment, the relative chlorophyll content in fully developed leaves was measured using a portable Konica SPAD-502 Plus meter (Konica Minolta Holdings Inc., Tokyo, Japan), with the results expressed as SPAD (Soil and Plant Analyzer Development) values. Morphological traits were determined as follows: plant height was measured with a ruler; fresh weights of shoots and roots were immediately measured using a SECURA2250-ICN balance (Gottingen, Germany); dry weights were obtained by drying the samples in an oven at 80°C for 48 h until a constant mass was achieved, followed by weighing with the same balance. Each treatment had three biological replicates, with five plants per replicate, and brown spots on leaves under Mn stress were documented by photography.

#### Metal element concentration and subcellular distribution

Leaf and root tissues were separately dried at 75°C for 4 days in an electric thermostatic drying oven (DHG-9123A, Shanghai, China) and then ground into a fine powder using a JC-FW100 grinder (Juchuan Co., Qingdao, China). For digestion, 100 mg of powdered plant material was accurately weighed into digestion tubes, followed by the addition of 4 mL concentrated nitric acid (HNO_3_) and 1 mL hydrogen peroxide (H_2_O_2_). The samples were digested in a graphite digestion system (ProD60, Kylin, Changsha, China) using a stepwise temperature program: 150°C for 10 min, 180°C for 10 min, and 200°C for 10 min. After digestion, residual acid was evaporated at 140°C on a BHW-09C heating block to a final volume of 0.5 mL. The digests were filtered and diluted to 50 mL with Millipore-purified water. Elemental (Mn, Ca, Mg, Fe, Zn, and Cu) concentrations were measured using inductively coupled plasma atomic emission spectrometry (ICP-AES, Juguang Co., Beijing, China) under the following conditions: RF power of 1.2 kW, plasma gas flow of 12 L/min (Ar), auxiliary gas flow of 1.0 L/min, nebulizer gas flow of 0.8 L/min, radial viewing mode, and a pump rate of 1.5 mL/min. Analytical wavelengths (nm) were 257.6 for Mn, 422.6 for Ca, 279 for Mg, 259 for Fe, 213.8 for Zn, and 324.7 for Cu. Certified reference materials (CRMs, Sigma-Aldrich, St. Louis, MO, USA) were used to prepare standard mixtures for instrument calibration and metal concentration quantification.

Cell sap preparation from root apices and Mn extraction from residual cell walls were performed following the modified protocol described by Wang.^57^ Specifically, root tip samples (1.0 g, approximately 1 cm in length) were loaded onto Ultra free-MC Centrifugal filter units (Millipore) and centrifuged at 3,000 × g for 10 min at 4°C to remove apoplastic solution. The roots were then subjected to three freeze-thaw cycles: −20°C for 8 h followed by thawing at room temperature for 30 min each. The cell sap fraction was isolated by centrifugation at 14,000 rpm for 15 min using an ultra-high-speed refrigerated centrifuge. Following centrifugation, the columns were rinsed three times with deionized water to ensure complete recovery of the fraction. For cell wall isolation, residual materials were ground to a fine powder under liquid nitrogen, homogenized in 75% ethanol, and centrifuged at 10,000 rpm for 10 min at 4°C. The pellet underwent sequential extractions with pre-cooled acetone, a methanol-chloroform (1:1, v/v) mixture, and methanol (three times for each solvent), followed by evaporation of organic solvents in a fume hood and drying to a constant weight at 65°C. Both cell sap and cell wall fractions were digested using microwave-assisted digestion prior to Mn quantification by ICP-AES.

#### Detection of root NA contents and exudation

After 6 days of Mn stress treatment, root exudates were collected according to the previously described method.^41^ Before collecting root exudates, the plants were removed from the nutrient solution, and the roots were rinsed 3 times with distilled water. Plant root systems were then immersed in 1 L of distilled deionized water for 4 h to collect exudates. The collected exudates were immediately filtered through filter paper (Whatman), concentrated to 2 mL in a vacuum evaporator at 35°C, and filtered through a 0.45 μm membrane filter (Millipore, Millex-HA). Samples were kept at −20°C until the analyses.

NA content was measured in wheat roots exposed to Mn stress. NA was analyzed using spectra obtained with liquid chromatography coupled with tandem mass spectrometry (LC-MS/MS) according to previously described methods.^41^ In brief, wheat roots (0.1 g) were homogenized in liquid nitrogen and extracted with 2 mL of 80% (v/v) methanol for 30 min under sonication at 25°C. The resulting extracts were filtered through a 0.45 μm membrane filter (Millipore, Millex-HA) and kept at −20°C until the analyses.

The contents of NA were determined via ultra-performance liquid chromatography-tandem mass spectrometry (UPLC-MS/MS, Agilent Technologies, Santa Clara, CA, USA) equipped with an Atlantis Premier BEH C18 AX column (2.1 × 100 mm, 1.7 μm; Waters, Milford, MA, USA). Chromatographic separation was achieved at 40°C using a binary mobile phase system with a flow rate of 0.3 mL/min. For electrospray ionization in positive mode (ESI+), the mobile phases consisted of 0.1% formic acid in 2% acetonitrile (A) and 0.1% formic acid in 100% acetonitrile (B); for negative mode (ESI−), 2% acetonitrile (A) and 100% acetonitrile (B) were used. The gradient elution program (total 6.3 min) was as follows: 99.5–5.0% A (0–4.0 min), isocratic 5.0% A (4.0–4.5 min), 5.0–99.5% A (4.5–4.8 min), followed by column re-equilibration (4.8–6.3 min). Authentic NA standards (Toronto Research Chemicals) were used to generate calibration curves, with quantification based on peak area normalization. Quality control was ensured through triplicate injections of three biological replicates, and results were expressed as mean ± SD.

#### RNA-seq analysis and RT-qPCR validation

Total RNA was extracted from wheat using TRIzol reagent (Invitrogen, Waltham, MA, USA). For RNA-seq analysis, three independent biological replicates were prepared. Total RNA samples were submitted to Shanghai Majorbio Bio-pharm Biotechnology Co., Ltd. (Shanghai, China) for library construction and sequencing on an Illumina HiSeq 2500 system (Illumina, San Diego, CA, USA). The raw RNA-seq reads have been deposited in the NCBI Sequence Read Archive (SRA) under BioProject accession number PRJNA1031207. Concurrently, bioinformatics analyses were performed on the company’s Majorbio Cloud comprehensive platform.^49^ The expression level of each transcript was calculated according to the fragments per kilobase of exons model per million mapped reads (FPKM) method. Gene abundances were further quantified using RSEM.^50^ Differential expression analysis was conducted with DESeq2.^51^ Genes exhibiting |log_2_FC| ≥ 1 and FDR ≤0.001 were defined as significantly differentially expressed genes (DEGs). Cluster heatmaps were generated using Toolkit for Biologists (TBtools, Ver. 2.030) with default settings.^52^ Additionally, Gene Ontology (GO) annotation and Kyoto Encyclopedia of Genes and Genomes (KEGG) pathway enrichment analyses of DEGs were performed using Goatools^53^ and KOBAS.^54^ Significantly enriched GO terms and KEGG pathways were identified based on a Bonferroni-corrected *p* ≤ 0.05 compared to the whole-transcriptome background.

For RT-qPCR validation, the first-strand cDNA was synthesized from the same total RNA used for RNA-seq analysis using the PrimeScript RT Master Mix. Real-time PCR reactions were pre-prepared by mixing TB Green Premix Ex Taq II (Takara Bio) with gene-specific primers designed with Primer3Plus (https://www.primer3plus.com/, listed in Table S2). Amplification was performed on the ABI 7500 system (Applied Biosystems, Waltham, MA, USA). Gene expression was quantified using the 2^−ΔΔCT^ method,^58^ with *TaActin* (accession number: XM_044554036.1) serving as the internal reference gene.

### Quantification and statistical analysis

Statistical analyses were performed using SPSS 20.0 (IBM Corp., Armonk, NY, USA). Data normality was assessed using the Shapiro-Wilk test (*p* > 0.05). Significant differences among groups were determined by one-way analysis of variance (ANOVA), followed by Duncan’s multiple range test (DMRT) for post hoc comparisons. Data are presented as mean ± standard deviation (SD), where n represents the number of independent biological replicates is provided for each experiment in the corresponding figure legend. The definition of statistical significance (*p* < 0.05) and the use of different lowercase letters to denote significant differences are also detailed in the figure legends.

## Supplemental information

Document S1. Figure S1 and Table S2

Table S1. Full list of DEGs and their functional annotation across wheat genotypes under manganese stress, related to Figure 1
