# Supplementary material for: Transcriptomic and physiological analyses revealed nicotianamine enhances wheat tolerance to excess manganese
Source: iScience. 2025 Oct 3;28(11):113671. doi: 10.1016/j.isci.2025.113671 (PMC12554150; doi:10.1016/j.isci.2025.113671)
Supplement: Document S1. Figure S1 and Table S2 [file mmc1.pdf]

iScience, Volume *28*

Supplemental information

Transcriptomic and physiological analyses revealed nicotianamine enhances wheat tolerance to excess manganese

Daozhen Luo (罗道祯), Qing Li (李青), Fei Pang (庞妃), Wenjie Zhang (张文洁), Muhammad Usman, Yangrui Li (李杨瑞), Yongxiu Xing (邢永秀), and Dengfeng Dong (董登峰)

Table S2. Sequences of primers used in qRT-PCR analysis, Related to Figure 2.

**Gene id (Gene name)**	**Forward primer (5'- 3')**	**Reverse primer (5'- 3')**
TraesCS6D02G148200 (NAS1-6B)	TTCCTGTACCCCATCGTCGA	GGCGACGATGACAGAGTTGA
TraesCS6D02G148600 (NAS1-6D)	TTCCTGTACCCCATCGTCGA	CGGGCGACAATGACAGAGTT
TraesCS2B02G060800 (NAS2-2B)	ACGTGCTTGCCAATGGACTT	TCACGTCCGTCACCATCTCA
TraesCS3B02G068500 (NAS2-3B)	ATGCATGCCAGTGGACTTCG	ACTTCGGCCTTGGCAAACTC
TraesCS4D02G184900 (NAS2-4D)	TGCCAGCTCCATCGATGAGT	AGGAGTGGAGTGGAGTTGGT
TraesCS5A02G552000 (NAS2-5A)	AGCACACCTCGGTACACACA	CACATGCACGTCCTTGGACT
TraesCS6A02G093000 (NAS4-6A)	ACAAGGCCAAGGTGATCGCA	GGACTTCTGCGCGATGATGA
TraesCSU02G125200 (NAS2-Un)	CCAAGGACGTGCATGTGAGT	ACCATCTCACCGAACCTGCA
TraesCS2A02G033500 (NAS5-2A)	TTCCTCGCGACATACCACCT	GCCAGGAAAACCACGTCGTA
TraesCS6A02G322500 (MTP8)	TCCTCGCCGTGTACACCATT	ATGGCGAGGTAGGTCAGCAT
TraesCS1D02G289700 (NAATA)	AACACAACGGCGATGCTCAT	TTCCGAGCTTTCTTGCCACC
TraesCS1A02G291200 (NAATB)	CCATGGCCGACAAGAACACA	TGCCGTATACCTCGTCAGCA
TraesCS7B02G227900 (Nramp1)	CGGTGTTCTCATTGCTGGCT	ACGAAGAAACATGCCGCCAT
TraesCS4A02G004400 (Nramp5)	TGTCGTTTGAGCTGCCGTTT	AGACGTTGATGCCGATGAGC
TraesCSU02G077000 (Nrat1)	TGGGGAATTTCAGGTGCAGC	AGACGACCCACAGAAGCTCA
TraesCS2B02G455100 (VIT1)	TGGGGAATTTCAGGTGCAGC	AGACGACCCACAGAAGCTCA
TraesCS5D02G209900 (VIT2)	TACATGTTCGTGGCGTCGGT	TAGCCGAAGAAAAGCAGCGC
TraesCS6D02G223000 (YSL2)	TGTGTGTGGGGAGCTTGGTA	CCCGGTGTGAACTTCATGCA
TraesCS2B02G301800 (YSL6)	CCCTTGGCTGTGTCATTGCT	CGCCCAAAATCGCCATCTCA
TraesCS2D02G387600 (YSL9)	ATTCTCGCGCTTGTCCCTCT	GCAAATCATCCCTGCCCCAA
TaActin	GCCCAGCAATGTATGTCGCA	ACCATCACCAGAGTCGAGCA

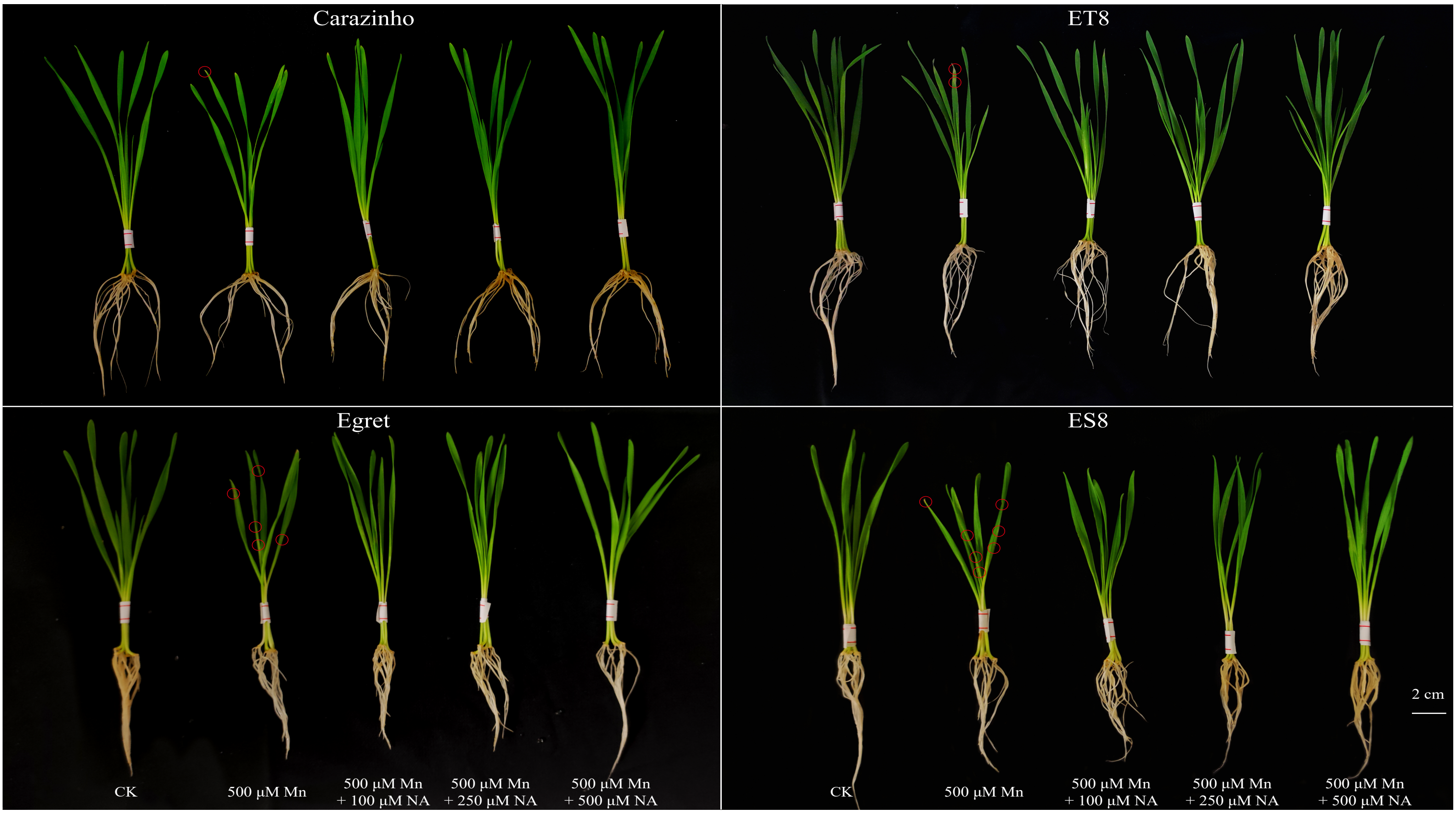

Figure S1. Visual symptoms of manganese toxicity and alleviation by exogenous nicotianamine (NA) across four wheat cultivars, Related to Figure 5.
Representative photographs of four wheat cultivars (Carazinho, Egret, ET8 and ES8) under Control, Mn stress (500 μM Mn), and Mn stress co-treated with increasing concentrations of NA (100, 250, 500 μM). Red circles highlight key symptoms of Mn toxicity. Exogenous NA application significantly alleviated these visual symptoms in a dose-dependent manner.
